# EXPERT BY EXPERIENCE WITH DEMENTIA CO-TEACHING: HAVING AN EXPERT IN EVERY CLASS

**DOI:** 10.1093/geroni/igae098.1937

**Published:** 2024-12-31

**Authors:** Michelle Kimzey

**Affiliations:** Texas Christian University, Fort Worth, Texas, United States

## Abstract

Care for people with dementia requires a better understanding of the reality of dementia. People with dementia are the true experts on dementia because they are living the experience. Including them in the teaching role benefits students and a person with dementia. The study aimed to understand how a person with dementia (expert by experience, [EBE]) involved in the teaching of an undergraduate dementia course impacted health profession students, an EBE with dementia, and their care partner. The qualitative exploratory design used semi-structured interviews for the student focus groups and interviews with an EBE and care partner. Students described the benefits of EBE, including increased awareness, knowledge, competencies, and reduced stigma. The EBE discussed the benefits, challenges, and meaningfulness of the experience. The care partner acknowledged the benefits for EBE and the family. Engaging people with dementia in EBE benefited students, families, and the EBE. By giving them a platform to educate students and self-advocate, we can positively impact the lives of people with dementia.

